# Supplementary material for: Cross-Resistance to Abiraterone and Enzalutamide in Castration Resistance Prostate Cancer Cellular Models Is Mediated by AR Transcriptional Reactivation
Source: Cancers (Basel). 2021 Mar 23;13(6):1483. doi: 10.3390/cancers13061483 (PMC8004828; doi:10.3390/cancers13061483)
Supplement: Supplementary file 1 [file cancers-13-01483-s001.pdf]

cancers

MDPI

Article

Cross-Resistance to Abiraterone and Enzalutamide in Castration Resistance Prostate Cancer Cellular Models is Mediated by AR Transcriptional Reactivation

Iris Simon [1,2], Sonia Perales [1,2], Laura Casado-Medina [1], Alba Rodríguez-Martínez [3,4], Maria del Carmen Garrido-Navas [3,5], Ignacio Puche-Sanz [6], Juan J. Diaz-Mochon [7,8], Clara Alaminos [9], Pablo Lupiañez [1,2], Jose A. Lorente [3,4], María J. Serrano [3,10,11,*] and Pedro J. Real [1,2,12,*]

[1] GENyO, Centre for Genomics and Oncological Research, Pfizer-University of Granada-Andalusian Regional Government, Gene Regulation, Stem Cells & Development Lab, PTS Granada, Avenida de la Ilustracion 114, 18016 Granada, Spain; iris.simon@genyo.es (I.S.); sopero@ugr.es (S.P.); lcasadom@idibell.cat (L.C.-M.); lupi13@correo.ugr.es (P.L.)

[2] Faculty of Science, Department of Biochemistry and Molecular Biology I, University of Granada, Avenida Fuentenueva s/n, 18071 Granada, Spain

[3] GENyO, Centre for Genomics and Oncological Research, Pfizer-University of Granada-Andalusian Regional Government, Liquid Biopsy and Cancer Interception Group, PTS Granada, Avenida de la Ilustracion 114, 18016 Granada, Spain; alba.rodriguez@genyo.es (A.R.-M.); carmen.garrido@genyo.es (M.d.C.G.-N.); jose.lorente@genyo.es (J.A.L.)

[4] Faculty of Medicine, Legal Medicine and Toxicology Department, University of Granada, Laboratory of Genetic Identification, Avenida de la Investigación 11, 18016 Granada, Spain

[5] Universidad Internacional de la Rioja, Avenida de la Paz, 137, 26006 Logroño, La Rioja, Spain

[6] Department of Urology, Bio-Health Research Institute (Instituto de Investigación Biosanitaria ibs.GRANADA), Hospital Universitario Virgen de las Nieves, University of Granada, Avenida de las Fuerzas Armadas 2, 18014 Granada, Spain; ignacio.puche.sspa@juntadeandalucia.es

[7] GENyO, Centre for Genomics and Oncological Research, Pfizer-University of Granada-Andalusian Regional Government, Nanochembio Lab, PTS Granada, Avenida de la Ilustracion 114, 18016 Granada, Spain; juanjose.diaz@genyo.es

[8] Department of Pharmaceutical and Organic Chemistry, Faculty of Pharmacy, Campus de Cartuja, University of Granada, 18071 Granada, Spain

[9] University Hospital of Jaen, Department of Urology, Avenida del Ejercito Español 10, 23007 Jaen, Spain; clararamosalaminos@juntadeandalucia.es

[10] Comprehensive Oncology Division, Clinical University Hospital, Virgen de las Nieves-IBS, Avenida de las Fuerzas Armadas 2, 18014 Granada, Spain

[11] Department of Pathological Anatomy, Faculty of Medicine, University of Granada, Avenida de la Investigación 11, 18016 Granada, Spain

[12] Bio-Health Research Institute (Instituto de Investigación Biosanitaria ibs.GRANADA), Personalized Oncology Group, Avenida de las Fuerzas Armadas 2, 18014 Granada, Spain

* Correspondence: mjose.serrano@genyo.es (M.J.S.); pedro.real@genyo.es (P.J.R.); Tel.: +34-958-715-500 (M.J.S. & P.J.R.)

Citation: Simon, I.; Perales, S.; Casado-Medina, L.; Rodr, A.; del Carmen Garrido-Navas, M.; Puche-Sanz, I.; Diaz-Mochon, J.J.; Alaminos, C.; Lupia, P.; Lorente, J.A.; et al. Cross-Resistance to Abiraterone and Enzalutamide in Castration Resistance Prostate Cancer Cellular Models is Mediated by AR Transcriptional Reactivation. *Cancers* **2021**, *13*, 1483. https://doi.org/10.3390/cancers13061483

Academic Editor: Daruka Mahadevan

Received: 1 February 2021
Accepted: 12 March 2021
Published: 23 March 2021

Publisher's Note: MDPI stays neutral with regard to jurisdictional claims in published maps and institutional affiliations.

Copyright: © 2021 by the authors. Licensee MDPI, Basel, Switzerland. This article is an open access article distributed under the terms and conditions of the Creative Commons Attribution (CC BY) license (http://creativecommons.org/licenses/by/4.0/).

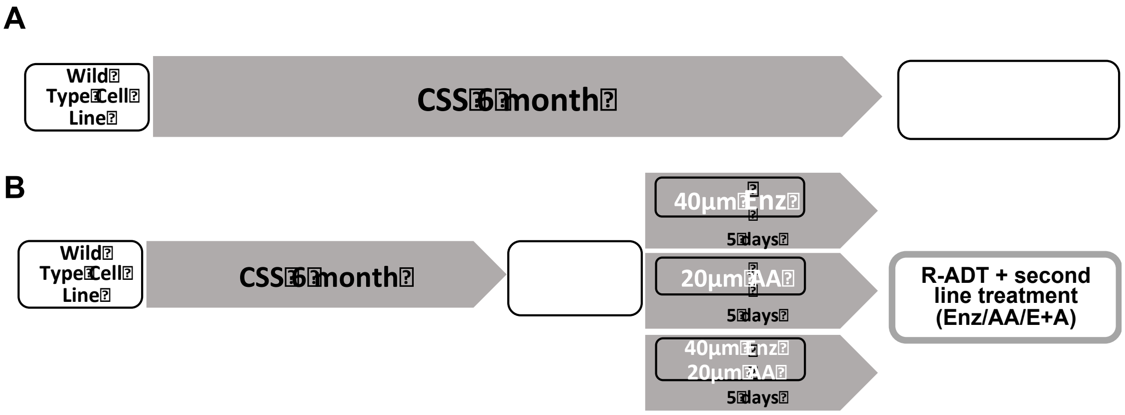

Figure S1. Development process of Resistance to ADT and establishment of the second line treatment (**A**) ADT Resistance development of LNCaP and 22RV1 cells lines (R-ADT). Wild-type cell lines, sensitive cells to ADT, were exposed to medium with charcoal stripped serum (CSS) for 6 months. (**B**) Second line treatment of R-ADT cells. R-ADT cells were treated with 40 µM Enzalutamide (Enz) (R-ADT +E), 20 µM Abiraterona (AA) (R-ADT + AA) or 40 µM Enz plus 20 µM AA (R-ADT+ E+A) for five days.

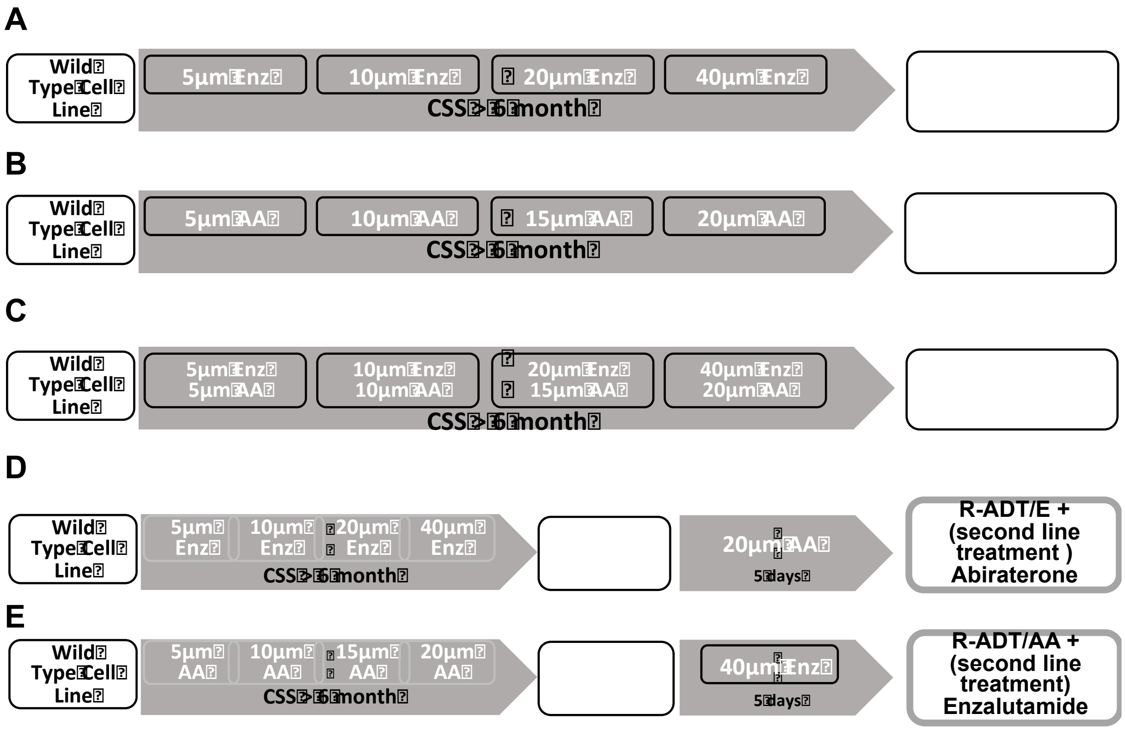

Figure S2. Development process of Resistance to ADT and new antiandrogens (Enz and/or AA) and establishment of the second line treatment. Novel Hormonal Agents (NHAs) resistance development of LNCaP and 22RV1 cells lines: (**A**) R-ADT/Enz; (**B**) R-ADT/AA and (**C**) R-ADT/E+AA. Treatment with Enz and/or AA was increased in a stepwise manner up to 4 times until reaching the highest final concentrations (20 µM for AA and 40 µM for Enz). All the cells were grown with charcoal stripped serum (CSS). Second treatment line of R-ADT/NHAs cells line: (**D**) R-ADT/E cells were treated with 20 µM AA for five days (R-ADT/E + AA); (**E**) R-ADT/AA cells were treated with 40 µM Enz (R-ADT/AA + ENZ) for five days.

A

ARV7

B

ARV9

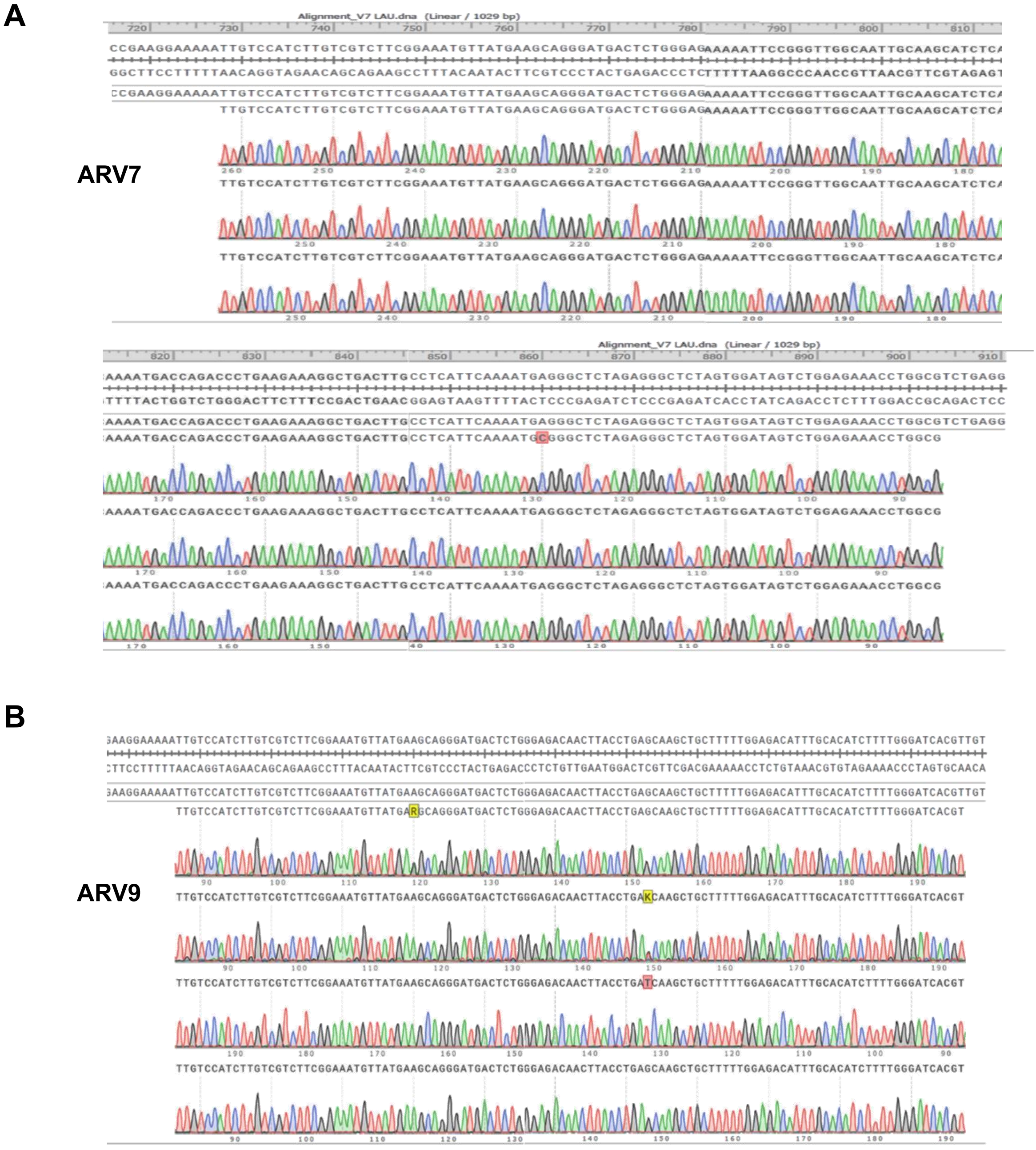

Figure S3. Alignment of the CDS of the *AR-V7* and *AR-V9* isoforms and the sequenced qPCR products. Examples of the positive alignments obtained between the cloned qPCR products. (**A**) Sequence of *AR-V7* and (**B**) sequence of *AR-V9*.

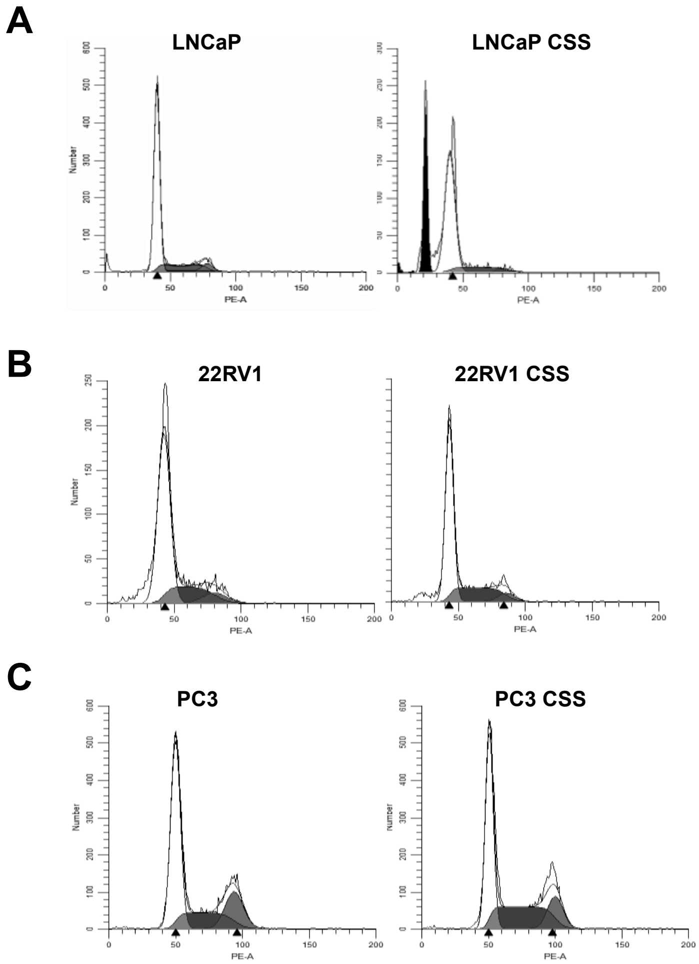

Figure S4. Cell cycle analysis with flow cytometry in wild-type PCa cell lines grown in ordinary medium and hormone-reduced medium (CSS). A representative cell cycle profile is included for each cell line and growth condition. Cells were stained with propidium iodide and cell cycle phases were determined using ModFit LT™. Every cell cycle phase was remarked in a different color: G_0/G_1 peak (white), G_2/M (Light grey), S (dark grey) and Sub-G_0 (black). (**A**) LNCaP wild-type cells. (**B**) 22RV1 wild-type cells. (**C**) PC3 wild-type cells.

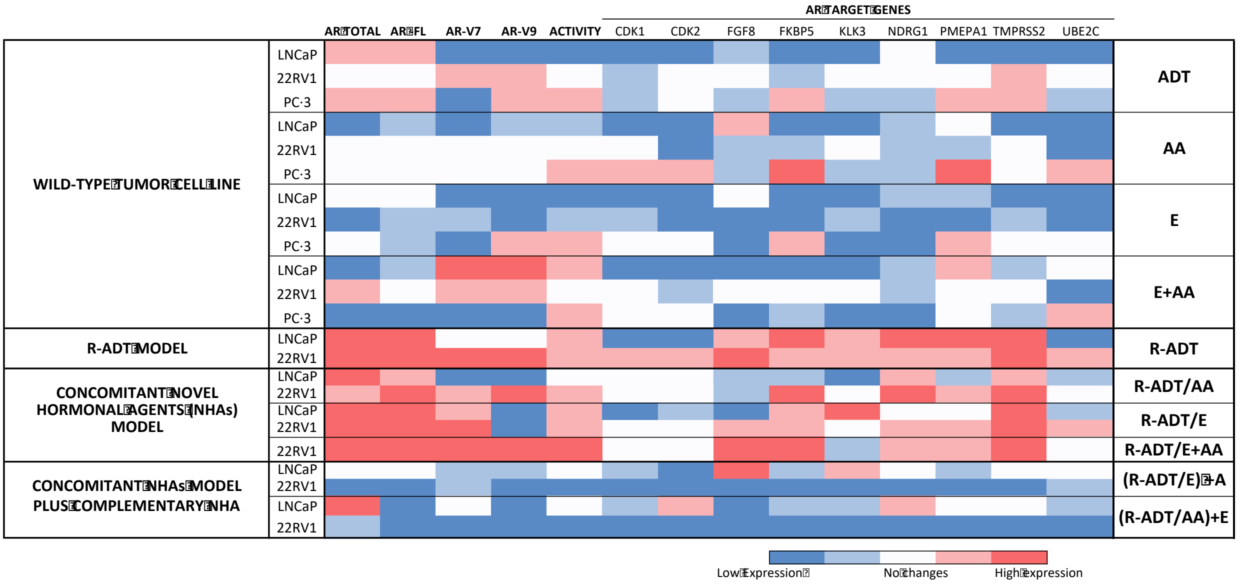

Figure S5. Heatmap representation of the expression levels of all the isoforms of AR (AR TOTAL), *AR full length*, *AR-V7* and *AR-V9* and their target genes in all cellular models. In addition to the transcription levels for AR, AR isoforms and AR target genes, we also included the concept of AR Activity (ACTIVITY) considering the average fold of the 9 AR target genes analyzed. The scale ranges from very low expression (less than 0.25-fold change), to low expression (from 0.25 to 0.5-fold change), no change (from 0.5 to 2-fold change), overexpressed (from 2 to 5-fold change) and highly overexpressed (more than 5-fold change).

Table S1. Primer list.

AR Total FW	5’-CCACTTGTGTCAAAAGCGAA-3’
AR Total RV	5’-AAGACCTGCCTGATCTGTGG-3’
AR full length FW	5’-TGTCCATCTTGTCGTCTTCG-3’
AR full length RV	5’-TTCAGATTACCAAGTTTCTTCAG-3’
AR-V7 FW	5’-TGTCCATCTTGTCGTCTTCG-3’
AR-V7 RV	5’-TAGTCTGGAGAAACCT-3’
AR-V9 FW	5’-TGTCCATCTTGTCGTCTTCG-3’
AR-V9 RV	5’-ACGTGATCCCAAAAGATGTG-3’
CDK1 FW	5’-TTTTCAGAGCTTTGGGCACT-3’
CDK1 RV	5’-CCATTTTGCCAGAAATTCGT-3’
CDK2 FW	5’-CATTCCTCTTCCCCTCATCA-3’
CDK2 RV	5’-CAGGGACTCCAAAAGCTCTG-3’
FGF8 FW	5’-GACCTACCAACTCTACAGCCG-3’
FGF8 RV	5’-CTCCTCGGACTCGAACTCTG-3’
FKBP5 FW	5’-TCCCTCGAATGCAACTCTCT-3’
FKBP5 RV	5’-AAACATCCTTCCACCACAGC-3’
KLK3 FW	5’-GTTGTCTTCCTCACCCTGTCC-3’
KLK3 RV	5’-GCAGCTGTGAGGACCCACT-3’
NDRG1 FW	5’-ACAACCCTGAGATGGTGGAG-3’
NDRG1 RV	5’-TGTGGACCACTTCCACGTTA-3’
PMEPA1 FW	5’-AAGATGCCCTGTCCTCAGAA-3’
PMEPA1 RV	5’-GTGCTGCAGGTACGGATAGG-3’
TMPRSS2 FW	5’-CACTGTGCATCACCTTGACC-3’
TMPRSS2 RV	5’-ACACGCCATCACACCAGTTA-3’
UBE2C FW	5’-ACCCAACATTGATAGTCCCTTG-3’
M13 FW	5’-GTAAAACGACGGCCAG-3’
